# *Plasmodium falciparum* resistance to artemisinin-based combination therapies

**DOI:** 10.1016/j.mib.2022.102193

**Published:** 2022-08-22

**Authors:** Kurt E Ward, David A Fidock, Jessica L Bridgford

**Affiliations:** 1 Department of Microbiology and Immunology, Columbia University Irving Medical Center, New York, NY 10032, USA; 2 Center for Malaria Therapeutics and Antimicrobial Resistance, Division of Infectious Diseases, Department of Medicine, Columbia University Irving Medical Center, New York, NY 10032, USA

## Abstract

Multidrug-resistant *Plasmodium falciparum* parasites are a major threat to public health in intertropical regions. Understanding the mechanistic basis, origins, and spread of resistance can inform strategies to mitigate its impact and reduce the global burden of malaria. The recent emergence in Africa of partial resistance to artemisinins, the core component of first-line combination therapies, is particularly concerning. Here, we review recent advances in elucidating the mechanistic basis of artemisinin resistance, driven primarily by point mutations in *P. falciparum* Kelch13, a key regulator of hemoglobin endocytosis and parasite response to artemisinin-induced stress. We also review resistance to partner drugs, including piperaquine and mefloquine, highlighting a key role for plasmepsins 2/3 and the drug and solute transporters *P. falciparum* chloroquine-resistance transporter and *P. falciparum* multidrug-resistance protein-1.

## Artemisinin-based combination therapies

Malaria continues to be a major global health problem, with an estimated 241 million cases and 627,000 deaths in 2020 [1]. *Plasmodium falciparum*, the most virulent causative species, accounts for ~98% of cases. Encouragingly, substantial reductions in the disease burden have been achieved over the past two decades, due in part to the implementation of artemisinin (ART)-based combination therapies (ACTs) as the first-line treatment for uncomplicated *P. falciparum* malaria in endemic countries worldwide.

ART and its derivatives are highly potent, fast-acting antimalarials that can reduce *P. falciparum* biomass by up to 10 000-fold every 48-hour asexual blood-stage cycle [2]. A three-day regimen of ART monotherapy is associated with high levels of parasite recrudescence due to a short plasma half-life (typically < 1–2 hr), thus necessitating the use of longer-lasting partner drugs in combination therapies. Partner drugs require a different mode of action, or mechanism of resistance, to reduce the chances of multidrug-resistance selection. The World Health Organization currently recommends six first-line ACTs: artemether-lumefantrine (AL), artesunate-amodiaquine (ASAQ), artesunate-mefloquine (AS-MQ), artesunate-sulfadoxine-pyrimethamine (AS-SP), dihydroartemisinin-piperaquine (DHA-PPQ) and artesunate-pyronaridine (AS-PND) [1]. AS-MQ and DHA-PPQ have been the predominant ACTs used in SE Asia, while AL and ASAQ are the main ACTs used in Africa and account for ~98% of doses delivered worldwide (Table 1).

One of the biggest roadblocks to malaria control is the ability of *P. falciparum* to rapidly evolve antimalarial resistance. ART-resistant *P. falciparum* emerged over a decade ago in the Greater Mekong Subregion (GMS), traditionally a hotbed for development of antimalarial resistance. Clinically, ART partial resistance is defined as delayed parasite clearance following artesunate monotherapy or ACT, and can be observed as a parasite-clearance half-life > 5 hr, or parasites microscopically evident on day 3 [3]. Initially, delayed clearance did not result in higher rates of treatment failure when artesunate monotherapy was followed with an effective partner drug [4]. Increased parasite exposure to partner drugs, however, led to the emergence of partner-drug resistance, notably to PPQ [5–9]. The resulting high rates of ACT treatment failure hindered control efforts in the GMS, leading to urgent calls for malaria elimination from this region to prevent the spread of multidrug-resistant parasites [10]. Malaria rates in this region have decreased substantially in recent years, and the greatest current concern is the independent emergence of ART partial resistance in sub-Saharan Africa, where 95% of malaria cases and deaths occur [1]. If ART partial resistance spreads across Africa and precedes partner drug and ACT failure, as witnessed in the GMS, the consequences could be devastating.

Here, we review recent research on resistance to ACTs and their components, including the multifaceted mechanisms underlying ART partial resistance, its geographic spread, resistance to DHA-PPQ in the GMS, and genetic modulators of partner-drug susceptibility.

## Artemisinin mode of action and mechanism of resistance

ART is activated by reduced heme iron (Fe^2+^ heme), which mediates cleavage of the endoperoxide bridge and results in the production of free-radical species (possibly carbon-centered [11]). These free radicals can alkylate heme, proteins, lipids, DNA, and other parasite biomolecules [12]. Widespread cell damage triggers the unfolded protein response through activation of protein kinase 4, leading to phosphorylation of eIF2α and subsequent inhibition of protein translation. This damage also leads to a buildup of polyubiquitinated proteins that are tagged for parasite proteasome-mediated degradation. This proteotoxic stress appears to be further ex-acerbated by ART-mediated inhibition of proteasome function, with studies showing synergy between ART derivatives and proteasome inhibitors [13–15]. Parasite responses to ART show similarities to heat-shock responses, and parasites unable to mount a heat-shock response display increased ART susceptibility [16,17]. ART derivatives may also inhibit the formation of hemozoin, leading to a buildup of Fe^2+^ heme [18]. This toxic by-product of parasite protease-mediated hemoglobin degradation is thought to be the primary activator of ART [19]. While disruption of the heme-degradation process decreases parasite sensitivity to ART, a recent study provided evidence that increasing heme levels also selectively antagonized ART’s antimalarial action, suggesting a multifactorial relationship between the heme-detoxification pathway and ART activation [20].

The primary genetic drivers of ART resistance, both *in vitro* and *in vivo*, are point mutations in *P. falciparum* K13 (also known as Kelch13), which reside mostly, although not exclusively, in the beta-propeller domain [21,22]. The causal relationship between several K13 mutations and resistance has been validated *in vitro* through reverse genetics [23–26]. K13 mutations allow a subset of early ring-stage parasites to survive cell-cycle arrest brought on by ART exposure, enabling those parasites to reinitiate transcription and complete their in-traerythrocytic developmental cycle once ART is no longer present at inhibitory concentrations [27–29•]. Resistance *in vitro* is routinely defined as > 1% survival of early ring-stage parasites exposed for 6 hr to 700 nM DHA (the primary active metabolite of ART) followed by drug-free culture incubation for a further 66 hr (this assay is referred to as the RSA_0–3 h_) [30]. These resistant parasites constitute a transcriptionally diverse population that differs in its expression of stress-response genes [31]. As discussed below, the mechanism of resistance appears to involve a complex interplay of K13 protein abundance, hemoglobin endocytosis, and the parasite response to stress.

K13-propeller mutations have been found in several studies to decrease the abundance of this essential protein, without altering *k13* transcription levels [27,32,33••]. This reduction is likely a result of altered protein-folding characteristics and solubility [34] and can differ by background and developmental stage [29•]. Downregulation of K13 protein levels results in decreased sensitivity to ART, and overexpression of either mutant or wild-type K13 resensitizes resistant parasites [33••–36].

K13 was recently localized to peripheral compartments in the parasite plasma membrane as well as vesicular compartments and the endoplasmic reticulum [25•,^32^,33••,^35^,^37^,^38^]. At the plasma membrane, K13 appears to be concentrated at the neck of hemoglobin-filled cytostomes that traffic the bulk of host hemoglobin from the red blood cell cytosol to the parasite’s lysosome-like digestive vacuole (DV) [32,33••]. Genetic mislocalization of K13 reduces cytostome trafficking and decreases the abundance of hemoglobin-derived peptides [32,33••]. Studies of K13-associated proteins have defined an interactome that includes multiple endocytosis proteins, including AP-2μ and the ubiquitin hydrolase UBP1, both of which have been linked to ART resistance [33••,^39^,^40^]. Conditional inactivation of several interactome proteins resulted in reduced ART susceptibility [33••]. These studies indicate a role for K13 in clathrin-independent endocytosis and hemoglobin uptake, suggesting that K13-mediated ring-stage resistance may result from reduced hemoglobin transport to the DV, and subsequent reduction of the drug activator Fe^2+^ heme.

Mutations in K13 also appear to mediate ART resistance through an increase in the baseline stress response, allowing for more effective mitigation of, or recovery from, the cell damage induced by ART exposure. The precise function of K13 is unknown, however, due to its sequence similarity to a class of Kelch/BTB/POZ ubiquitination adapters, it has been postulated that K13 acts as a ubiquitin ligase adapter that mediates ubiquitin-dependent targeting of proteins for proteasomal degradation [19]. Several analyses have associated K13 mutations with upregulation of chaperones and pathways involved in the unfolded protein response and oxidative stress, and downregulation of ubiquitinating enzymes [25•,^27^,29•,^41^]. These factors, in addition to reduced hemoglobin uptake, may explain lower levels of oxidative stress observed in ART-resistant parasites, both at baseline and in response to ART exposure [42]. Increased survival of K13 mutant parasites following ART-induced dormancy may also involve increased baseline phosphorylation of eIF2α and extended ring-stage development [13,32].

Several other interesting phenotypes have been observed in ART-resistant parasites. Enhanced DNA damage repair pathways associated with K13 mutation may aid recovery from ART-induced DNA damage [43]. Mitochondrial proteins are also heavily involved in the ART stress response [44,45], and mutant K13-mediated resistance can be reversed by the mitochondrial electron-transport chain inhibitor atovaquone [29•]. K13 mutant parasites have also been observed to asynchronously reinitiate growth 18–24 hr after removal of DHA pressure, a feature that may be separate from the effect of mutant K13 on restricting hemoglobin endocytosis in ring stages, raising the question of how parasites emerge from their state of ART-induced quiescence [29•].

The degree of protection to ART offered by K13 mutations varies considerably by parasite background, suggesting that secondary determinants contribute to resistance [25•,^26^,^46^]. Gene-editing studies recently provided evidence of a greater fitness cost resulting from the introduction of K13 mutations into African strains compared with Asian strains, suggesting that the latter have additional genetic factors that compensate for mutant K13 fitness costs [26]. Mutations in several other proteins have been reported to mediate *in vitro* resistance to ART, several of which (PI3P, AP-2μ, UBP1, and KIC7) have been colocalized or associated with K13 [33••], and others that are also thought to play a role in vesicular trafficking (coronin, falcipain 2) [19,21,47]. These studies underline the importance of research into K13 and other potential ART-resistance mediators, particularly in light of the recent emergence of resistance in Africa [24••,^48^–^50^].

## The spread of mutant K13-driven artemisinin partial resistance

ART partial resistance and causative K13 mutations are now widespread across the GMS, which together with partner-drug resistance, has resulted in high ACT failure rates [1]. Over 200 nonsynonymous K13 mutations have been identified in parasite populations globally, of which 12 are validated to confer ART resistance and 10 are associated with resistance (Table 2) [3,21]. The C580Y allele dominates most of the GMS, with the exception of Myanmar where the F466I allele is more common [51•]. The C580Y allele also emerged independently in Guyana and Papua New Guinea, but at low frequency and with no apparent effect as yet on ACT efficacy [52,53].

Initial signs of ART partial resistance are now emerging in sub-Saharan Africa. The K13 mutation R561H, which is a validated marker of resistance in the GMS, emerged independently in Rwanda and has rapidly attained a prevalence of 22% in some sites [24••,^50^]. R561H was significantly associated with day-3 parasitemia [48] and a longer parasite-clearance half-life following AL treatment [50]. Gene editing has shown that R561H can confer a degree of *in vitro* ART partial resistance similar to that of C580Y and is fitness-neutral [26], indicating that the Rwandan R561H lineage has the potential to predominate in this region.

A recent study from northern Uganda reported an increased prevalence of the K13 mutations C469Y and A675V, attaining 23% and 41%, respectively [54]. Both mutations were associated with delayed parasite clearance following intravenous artesunate monotherapy [49••]. Although A675V demonstrated *ex vivo* ART resistance [49••], both C469Y and A675V mediated either no significant shift, or only a modest increase in ring-stage survival in a subsequent gene-editing study [46], highlighting the importance of the parasite genetic background in mutant K13-mediated resistance. Continued surveillance across Africa of K13 polymorphisms, parasite-clearance times, and *in vitro* partial resistance will be extremely important in the coming years.

## *P. falciparum* chloroquine-resistance transporter and *P. falciparum* multidrug-resistance protein-1 as modulators of susceptibility to artemisinin combination therapies partner drugs

ASAQ and AS-MQ have been used extensively in the GMS, and high failure rates to both have been reported [3,55•]. Artesunate-pyronaridine (AS-PND), the most recently deployed ACT, shows excellent efficacy against DHA-PPQ-resistant infections [56]. AL is the predominant first-line ACT in Africa, and there is no concrete evidence of lumefantrine (LMF) or pyronaridine (PND) resistance. The 4-aminoquinolines amodiaquine (ADQ) and PND act primarily by inhibiting hemozoin formation in the DV, whereas the arylaminoalcohols mefloquine (MFQ) and LMF only partially inhibit hemozoin formation, with their primary targets thought to be located in the cytosol [21,57].

Resistance to ACT partner drugs is mostly associated with point mutations in the *P. falciparum* chloroquine-resistance transporter (*pfcrt*) and *P. falciparum* multidrug-resistance protein-1 (*pfmdr1*) genes that encode two DV membrane transporters, or amplification of *pfmdr1* (Table 2). These mutations often have opposing effects on susceptibilities to partner drugs. Treatment with ASAQ selects for PfCRT K76T, a mutation critically required for chloroquine (CQ) resistance, and PfMDR1 N86Y, a modulator of CQ resistance, while AL treatment selects for the wild-type alleles [58,59]. *In vitro*, PfMDR1 N86Y reduces susceptibility to ADQ and PND and increases susceptibility to LMF, MFQ, and DHA [60,61]. Additionally, *pfmdr1* amplification is the most important determinant of MFQ resistance in Southeast Asia [62,63].

**Figure 1:**
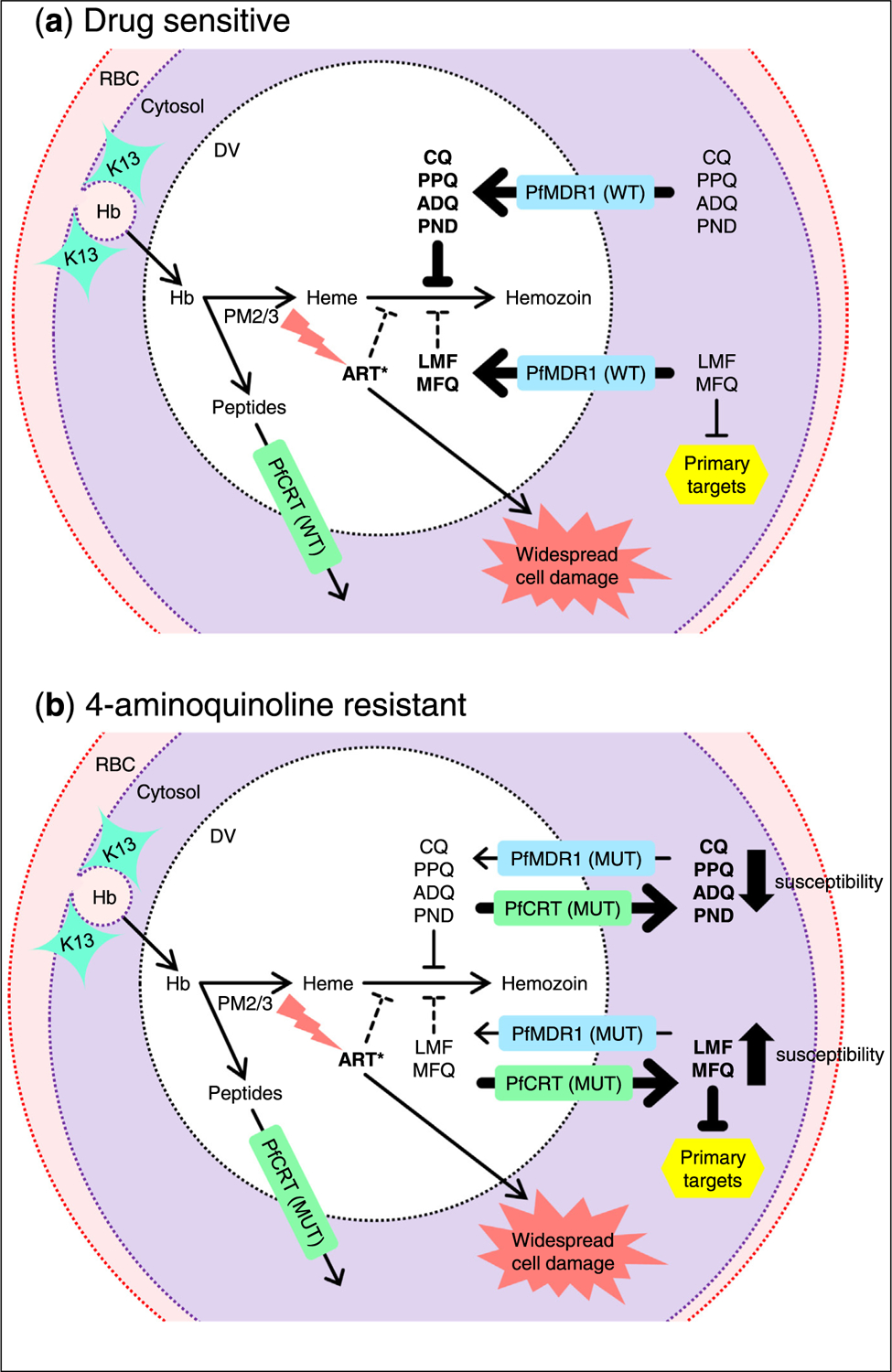
Model of select antimalarial drug modes of action and resistance determinants. **(a)** K13 is involved in endocytosis of hemoglobin-containing host cytosol and trafficking to the DV, where hemoglobin is degraded by parasite proteases, including plasmepsins 2/3 to release peptides and heme. Fe^2+^ heme activates ART derivatives *via* cleavage of their endoperoxide bridge. The 4-aminoquinolines (CQ, PPQ, ADQ, and PND) act primarily by inhibiting biomineralization of toxic heme to inert hemozoin. LMF, MFQ, and ART derivatives partially inhibit hemozoin formation and are thought to act on primary targets in the cytosol. PfMDR1 (WT) transports diverse compounds from the cytosol into the DV. PfCRT (WT) does not mediate drug transport, but transports globin-derived peptide residues from the DV to the parasite cytosol. **(b)** Mutations in PfMDR1 and PfCRT (MUT) are thought to decrease and confer drug-transport capacity, respectively, reducing drug accumulation in the DV and increasing concentration in the cytosol, resulting in 4-aminoquinoline resistance and increased susceptibility to LMF, MFQ, and ART. Hb, hemoglobin; RBC, red blood cell.

One endogenous function of PfCRT appears to be proton-dependent transport of hemoglobin-derived peptides out of the DV [64,65], while PfMDR1 is predicted to transport solutes into the DV. Wild-type PfMDR1 has been reported to transport diverse pharmacophores, including LMF, MFQ, DHA, PPQ, ADQ, and CQ, and PfMDR1–86Y reduces transport of these compounds [66•]. In contrast, wild-type PfCRT cannot efflux these compounds, and only drug-resistant PfCRT isoforms transport CQ, LMF, and MFQ [66•]. As a result, mutant isoforms of PfCRT and PfMDR1 are thought to decrease accumulation of antimalarials in the DV and increase their concentration in the cytosol, thereby conferring altered drug susceptibility based on the location of the drug targets (Figure 1).

The collateral drug sensitivity of *pfcrt* and *pfmdr1* alleles is reflected in changes of allele frequencies in response to replacement of first-line therapies. The prevalence of multicopy *pfmdr1* in the GMS decreased dramatically upon withdrawal of AS-MQ [67,68]. The prevalence of *pfcrt-*76T and *pfmdr1-*86Y, which increased in Sub-Saharan Africa under CQ pressure, decreased dramatically from 2004 to 2018 in response to widespread uptake of AL [54,69,70]. Worryingly, *ex vivo* parasites recently collected from patients in Eastern Uganda had a small but significant decrease in susceptibility to LMF, which was accompanied by a significant increase in the prevalence of *pfmdr1*-N86 to > 99% [71•]. This suggests an increasing prevalence of LMF-tolerant parasites, which is concerning, given the recent emergence of ART partial resistance in east Africa.

## Piperaquine resistance associated with multiple plasmepsins 2/3 and novel mutations in *P. falciparum* chloroquine-resistance transporter

DHA-PPQ replaced AS-MQ as the first-line ACT in Cambodia in the midst of emerging ART resistance, and within a few years, high treatment-failure rates were reported [3]. Multiple genome-wide association studies identified amplification of *plasmepsins 2* and *3* (*pm2/3*) and novel point mutations in *pfcrt* as being associated with clinical and *in vitro* PPQ resistance (Table 2) [21]. Multicopy *pm2/3* was associated with increased parasite survival at elevated PPQ concentrations [72]. However, *pm2* overexpression alone failed to confer *in vitro* PPQ resistance [73]. Conversely, gene editing validated that novel PfCRT point mutations can drive high-grade *in vitro* PPQ resistance, including on a *pm2/3* single-copy background [74]. Longitudinal surveillance revealed a rapid increase in the prevalence of these PfCRT mutations following DHA-PPQ implementation, with > 98% of parasites harboring these mutations by 2017 [67]. Outside the GMS, a novel PfCRT variant causal for PPQ resistance emerged independently in French Guiana [75]. In China, emergence of novel PfCRT haplotypes conferring *in vitro* PPQ resistance may explain early PPQ clinical failures in this region [76].

PfCRT mutations that confer PPQ resistance can partially or fully reverse CQ and ADQ resistance based on the isoform on which they arise [74,76,77]. PPQ, a 4-aminoquinoline, exerts its antimalarial action by inhibiting heme detoxification in the DV [77]. Studies of PfCRT-mediated drug transport demonstrate that CQ-resistant isoforms, which have a large transport capacity for CQ, do not transport PPQ [78]. Conversely, PPQ-resistant isoforms efflux PPQ and reduce CQ transportation, leading to increased CQ accumulation in the DV [76].

## Conclusions and future outlook

Recent years have seen a tremendous increase in research into the molecular basis of antimalarial drug resistance in *P. falciparum* parasites, complementing parallel efforts to identify new drug targets and candidate medicines. These efforts provide hope that within the next decade, we can substantially decrease the burden of disease, especially in high-transmission settings in Africa. However, the recent detection in eastern Africa of mutant K13 parasites is particularly worrying, as it may lead to partner-drug resistance and ACT- treatment failures, as earlier occurred in the GMS in south-east Asia. To date, AL remains broadly effective across Africa. Detailed monitoring for resistance, both genotypically and measuring parasite susceptibility, is essential. Genetic crosses to map genetic determinants of resistance, and gene-editing methods to establish causality, provide powerful tools to characterize resistance [79,80]. Mathematical modeling also provides a key approach to optimize treatment and control measures at a national and subnational level [81–83]. Several new approaches are also being actively explored, including triple ACTs, or drug-rotation strategies with multiple ACTs, which could exploit the opposing selective pressures that partner drugs place on PfCRT and PfMDR1 as mediators of parasite susceptibility [2,84•]. These efforts extend to chemoprevention measures such as seasonal malaria chemoprevention or intermittent preventive treatments, whose efficacy can also be optimized through a detailed understanding of the genetic and molecular basis of antimalarial resistance [85]. The exceptional coordination between scientists in academia and industry, supported by multiple organizations including the Bill & Melinda Gates Foundation, the Medicines for Malaria Venture, the World Health Organization, and governmental agencies and science-based funders, is a key foundation for increasing efforts to regain the upper hand against malaria and dramatically reduce its global impact.

## Figures and Tables

**Table 1:** Artemisinin-based combination therapies recommended by the WHO as first-line treatments for uncomplicated *P. falciparum* malaria.

ART derivative	Partner drug	Abbreviation	World use	Regions where first-line				
Artemether	Lumefantrine	AL	74%	AFRO	AMRO	SEARO	EMRO	WPRO
Artesunate	Amodiaquine	ASAQ	24%	AFRO				
Dihydroartemisinin	Piperaquine	DHA-PPQ	1%	AFRO		SEARO		WPRO
Artesunate	Pyronaridine	AS-PND	0.1%			SEARO		WPRO
Artesunate	Mefloquine	AS-MQ	1%		AMRO	SEARO		WPRO

Artesunate-sulfadoxine-pyrimethamine (AS-SP) is also a recommended first-line ACT in EMRO and SEARO. WHO regions: AFRO, African Region; AMRO, Region of the Americas; SEARO, South-East Asia Region; EMRO, Eastern Mediterranean Region; WPRO, Western Pacific Region.

**Table 2:** Molecular markers of antimalarial drug resistance in *P. falciparum.*

Class	Drug	Gene (s)	Mutation
4-aminoquinolines	Chloroquine	*pfcrt*	Necessary but not sufficient: K76T^a^ Other mutations include C72S, M74I, N75E, A220S, Q271E, N326S, I356T, and R371I
		*pfmdrl* (in combination with mutant *pfcrt* only)	N86Y, Y184F, S1034C, N1042D, and D1246Y
	Amodiaquine	*pfcrt* and *pfmdrl*	PfMDR1 N86Y and PfCRT K76T contribute to decreased parasite susceptibility
	Piperaquine	*pm2/3*	Increased copy number
		*pfcrt*	T93S, H97Y, F145I, I218F, M343L, C350R, or G353V
	Pyronaridine	Yet to be identified	-
Amino alcohols	Lumefantrine	Yet to be identified	Selects for PfMDR1 N86 and PfCRT K76
	Mefloquine	*pfmdrl*	Increased copy number
Endoperoxide	Artemisinin derivatives	*k13*	Validated: P413A, F446I, N458Y, C469Y, M476I, Y493H, R539T, I543T, P553L, R561H, P574L, C580Y, and A675V^b^ Candidate or associated: P441L, G449A, C469F, A481V, R515K, P527H, N537I/D, G538V, and R622I

a Chloroquine resistance requires at least four mutations that include K76T.

b Validated K13 markers have a statistically significant association between the mutation and delayed clearance as well as survival > 1% in the ring-stage survival assay. Candidate or associated markers fulfill one of these conditions to date.
